# Sleep satisfaction, and its correlates with stress, health and happiness in university students: cultural and gender issues

**DOI:** 10.1192/j.eurpsy.2024.1613

**Published:** 2024-08-27

**Authors:** E. L. Nikolaev, S. S. Fakhraei, T. Nikolaeva

**Affiliations:** ^1^Medical Faculty; ^2^Department of Social and Clinical Psychology, Ulianov Chuvash State University, Cheboksary, Russian Federation

## Abstract

**Introduction:**

Sleep plays an important role in preserving mental health. University students’ learning activity, habits and cultural background may negatively affect the duration and quality of sleep.

**Objectives:**

To determine the correlations of sleep satisfaction with the level of stress, health and happiness in university students of different gender and cultural backgrounds

**Methods:**

We have surveyed 134 university students (77 domestic students and 57 foreign students). The numbers of male and female students were the same (67 students).To determine the levels of stress, health, happiness, and sleep quality satisfaction, we used a self-rating questionnaire (Nikolaev, 2023).

**Results:**

The general indicator of sleep satisfaction with all the respondents made up 6.22±2.4 points. We have not revealed any valid statistic differences between the satisfaction levels of males and females, domestic and foreign students (p>.05). The males have shown a higher level of stress than females (р=.0004). The higher level of health assessment was revealed by foreign students as compared with domestic students (р=.0137), and by males in comparison with females (р=.0.0054). We did not determine any cultural and gender differences in other parameters. (p>.05). According to the final correlation analysis, all the respondents showed that their level of sleep satisfaction was positively correlated with the level of health (r=.40) and happiness (r=.37), but negatively with the level of stress (r=-.23). Similar interrelations were seen in the male group (r=.40; r=.36; r=-.28). Females revealed correlations of their sleep satisfaction with health (r=.38) and happiness (r=.38), but there was no evidence of correlation with the level of stress (p>.05).

**Conclusions:**

University health development programs aimed at improving their students’ sleep quality, which take into account the complex of cultural and gender issues, may help enhance the students’ health potential.

**Disclosure of Interest:**

None Declared

